# The traditional Chinese medicine formula Zhihan Anshen Tang (ZHAST) against obstructive sleep apnea hypopnea syndrome: network pharmacology and molecular docking approach

**DOI:** 10.3389/fchem.2025.1524087

**Published:** 2025-03-10

**Authors:** Cai-Li Li, Yu-Xiang Zhang, Xing-Jie Zheng, Shuo Li, Jing Feng

**Affiliations:** ^1^ Respiratory Department, Tianjin Medical University General Hospital, Tianjin, China; ^2^ Infectious Disease Department, Tianjin Haihe Hospital, Tianjin, China

**Keywords:** obstructive sleep apnea hypopnea syndrome, Zhihan Anshen Tang, network pharmacology, molecular dynamics simulation BBB, signaling pathway

## Abstract

**Introduction:**

The current treaments for Obstructive Sleep Apnea Hypopnea (OSAHS) are Continuous Positive Airway Pressure (CPAP) and lifestyle modifications, which is not suitable for all patients. Traditional Chinese medicine (TCM) has increasingly demonstrated its efficacy and benefits in treating OSAHS. Zhihan Anshen Tang (ZHAST), has been demonstrated its efficacy and clinical metrics for treating OSAHS patients. However, its key ingredients and mechanisms of action are still unknown.

**Methods:**

Using network pharmacology, we investigated the potential mechanisms of ZHAST through which OSAHS.

**Results:**

In addition, the key targets, including TNF, IL6, GAPDH, STAT3, HIF1A, and JUN, are revealed by the topological analysis. According to the findings of the GO enrichment analysis, genes were enriched in inflammatory responses, hypoxia responses, positive regulation of angiogenesis, protein phosphorylation, and regulation of cell proliferation. KEGG pathway enrichment analysis suggests that the signaling pathway of ZHAST in OSAHS are MAPK and AGE-RAGE signaling pathway, especially in diabetic complications. In addition, it is demonstrated that the enoxolone in ZHASTs have high affinity with the relevant targets by molecular docking and molecular dynamics simulations.

**Disscussion:**

To my knowledge, this is the first network pharmacological molecular docking study about a Chinese medicine effective against OSA. This investigation integrates molecular docking and network pharmacology to identify the effective compounds, related targets, and potential mechanism of ZHASTs in the treatment of OSAHS, providing the prospect of traditional Chinese medicines with modern medical research.

## 1 Introduction

Obstructive Sleep Apnea Hypopnea Syndrome (OSAHS) is a common clinical sleep disorder, categorized in traditional Chinese medicine under the terms “hypersomnia” and “snoring” (Zhou et al., 2023). It is primarily characterized by snoring, apneas, and hypopnea during sleep, along with disrupted sleep architecture and recurrent hypoxemia (Zhou et al., 2023; Lv et al., 2023). These conditions frequently result in fragmented sleep, arousals caused by snoring, daytime somnolence, and dizziness. They can predispose patients to cardiovascular, cerebrovascular, pulmonary diseases, and multi-organ damage severely impacting their quality of life and lifespan (Chen et al., 2021; Di Bello et al., 2023; Lebkuchen et al., 2021).

In recent years, traditional Chinese medicine (TCM) has increasingly demonstrated its efficacy and benefits in treating OSAHS. TCM can effectively alleviate symptoms, control disease progression, and enhance sleep functionality and quality of life. Zhihan Anshen Tang (ZHAST), a classic traditional Chinese medicinal formula, comprises the following ingredients: Ban Xia, Chen Pi, Zhi Shi, Zhu Ru, Shi Chang Pu, Yuan Zhi, Huo Xiang, Yu Jin, Dan Shen, Niu Bang Zi, Fu Shen, Ye Jiao Teng, Chao Zao Ren, Bai Zhi, Hong Jing Tian, Hai Fu Shi, Qing Meng Shi, Dan Nan Xing, and Zhi Gan Cao. Clinical studies have demonstrated that ZHAST significantly improves therapeutic outcomes and clinical metrics in patients with OSAHS. Previous research has confirmed that Ban Xia (Song et al., 2022), as one of the traditional Chinese medicines, has shown potential therapeutic effects in recent studies. In the treatment of patients with OSAHS accompanied by insomnia, Zhihan Anshen Tang has demonstrated unique therapeutic efficacy.

Network pharmacology, as an emerging interdisciplinary field, is increasingly becoming an essential tool for unraveling the complexities of mechanisms in traditional Chinese medicine (Wang y. et al., 2024). This discipline utilizes modern techniques such as database filtering, computer simulation, and data mining to effectively identify key therapeutic targets and predict associated signaling pathways and mechanisms of action. Not only does this aid in a deeper understanding of the biological nature of complex diseases and the molecular actions of drugs, but it also accelerates the discovery of new biomarkers and the development and clinical application of new drugs. Through these methods, network pharmacology offers an efficient approach to optimizing drug research and therapeutic strategies. For example, A. Bisht and colleagues integrated network pharmacology, molecular docking, and molecular dynamics simulation to elucidate the anti-aging mechanism of Tinospora cordifolia (Bisht et al., 2024). Their research not only identified key active compounds but also confirmed their stability through molecular dynamics simulations, offering new perspectives for the development of anti-aging drugs. Similarly, Gu et al. (2024) and colleagues employed network pharmacology and multi-omics analysis to explore the active components and mechanisms of Si Ni Tang in treating sepsis. They discovered that by modulating signaling pathways related to oxidative stress and lipid metabolism, Si Ni Tang could improve the prognosis for sepsis patients. Furthermore, Li et al. (2024) and others utilized network pharmacology to analyze the potential mechanisms of Jian Gu Granules in treating postmenopausal osteoporosis. These studies demonstrate that network pharmacology not only aids in understanding the complex mechanisms of traditional Chinese medicine but also guides the development and clinical application of new drugs. With ongoing advancements in technology, it is reasonable to believe that network pharmacology will play an increasingly significant role in future research on traditional Chinese medicine.

**FIGURE 1:**
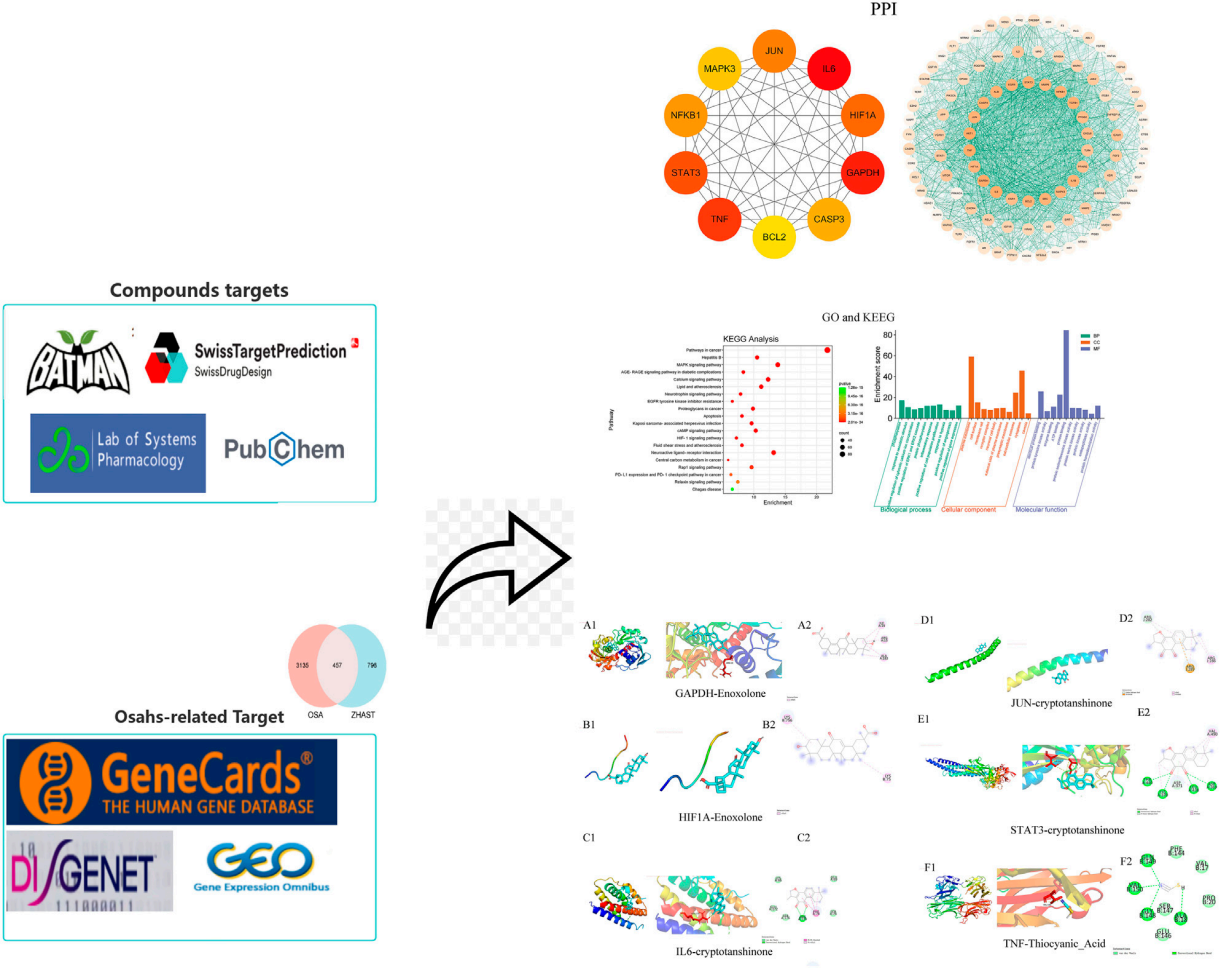
Workflow of the network pharmacological investigation strategy of ZHAST in the treatment of OSAHS.

Despite numerous fundamental studies being conducted, the specific targets and precise mechanisms involved in treating OSAHS with ZHAST still remain unclear. In this study, we utilized bioinformatics methods to examine the primary chemical components of ZHAST along with their potential targets and mechanisms for addressing OSAHS. This research establishes a theoretical foundation for further investigating the pharmacodynamics, material composition, and modes of action associated with using ZHAST to treat OSAHS. The research flowchart is depicted in Figure 1.

## 2 Materials and methods

### 2.1 data collection

#### 2.1.1 Selection of active compounds and therapeutic targets for ZHAST

The active ingredients in ZHAST were identified and selected from both the TCMSP database (RU et al., 2014) (https://old.tcmsp-e.com/tcmsp.php) and the BATMAN database (Kong et al., 2024) (http://bionet.ncpsb.org.cn/batman-tcm/#/home), using criteria of OB ≥ 30 and DL ≥ 0.18 (Van Der Graaf and Benson, 2011). Subsequently, these identified active compounds were queried in the PubChem database (Wang et al., 2012a) (https://pubchem.ncbi.nlm.nih.gov/) to obtain their SMILES notation, which was then entered into the SwissTargetPrediction database(http://www.swisstargetprediction.ch/) for target prediction.

#### 2.1.2 Establishment of a database for OSAHS targets

Using the GEO database (https://www.ncbi.nlm.nih.gov/gds) with ‘Obstructive sleep apnea hypopnea’ as the search term, we selected dataset GSE38792. Batch effects were eliminated using the limma package, and differentially expressed genes were identified based on selection criteria of |logFC| > 1 and P < 0.05. Heatmaps were generated utilizing the ggplot2 package. Furthermore, disease targets associated with obstructive sleep apnea were queried through Genecards (https://www.genecards.org) (Safran et al., 2002), OMIM (https://www.omim.org) (Landrum et al., 2016), and DisGenet (https://www.disgenet.org) databases (Piñero et al., 2020), thus establishing a comprehensive database of targets relevant to OSAHS.

### 2.2 Establishment of PPI network

The Venny 2.1.0 tool (https://bioinfo.cnb.csic.es/tools/venny/index.html) was used to analyze the overlaps between the predicted ZHAST targets and OSAHS-associated targets. To obtain PPI data, common targets between the drug and disease were inputted into the STRING database (Szklarczyk et al., 2023) (https://string-db.org/cgi/input.pl) for constructing the PPI network, with species set as “*Homo sapiens*” and a minimum required interaction score of 0.40. The resulting network was visualized using Cytoscape (Otasek et al., 2019), where key targets were identified based on topological parameters such as Degree Centrality (DC), Betweenness Centrality (BC), and Closeness Centrality (CC). The selection criteria for these key targets were values greater than twice the median value. Additionally, to identify central targets, we utilized the Cytohubba plugin in Cytoscape which calculates Maximum Clique Centrality (MCC). Furthermore, module filtering within the PPI network was performed using the MCODE plugin in Cytoscape for cluster analysis (Xie et al., 2022).

### 2.3 Selection and analysis of key targets

Core genes were identified through topological analysis of the PPI network and the MCC algorithm in the Cytohubba plugin. Gene and protein expressions in adipose tissue were obtained from the Human Protein Atlas (HPA, https://www.proteinatlas.org/) (Zhou et al., 2022). This approach enables a systematic analysis, facilitating a deeper understanding of gene expressions relevant to the study’s focus on adipose tissues.

### 2.4 GO and KEGG enrichment analyses

Shared drug-disease targets were uploaded to DAVID Bioinformatics Resources 6.8 (https://david.ncifcrf.gov/home.jsp) for GO enrichment analysis, encompassing biological processes (BP), cellular components (CC), and molecular functions (MF). Enrichment thresholds were set at PvalueCutoff = 0.05 and QvalueCutoff = 0.05, with default settings used otherwise (Ding and Liu, 2023). Additionally, KEGG pathway enrichment analysis was conducted on these targets by selecting entries with an adjusted P-value <0.05. The top 10 GO terms and top 20 KEGG pathways were visually represented based on the significance of enrichment, providing valuable insights into the molecular interactions and pathways involved.

### 2.5 Network construction

The herbal-compound-target (H-C-T) network was constructed using Cytoscape 3.7.2, with traditional Chinese medicine serving as the foundation for identifying active compounds and shared targets. Topological analysis was also conducted utilizing the same software. The formation of the H-C-T network is based on the inclusion of active compounds found in ZHAST and their corresponding shared targets. To enhance our understanding of pathway relationships, compounds, and targets, we prioritized the top 20 pathways along with their associated targets and compounds to construct a Compound-Target-Pathway (C-T-P) network.

### 2.6 ADME and toxicity predictions

The ADMET properties, encompassing absorption, distribution, metabolism, excretion, and toxicity evaluation of ten selected drugs were assessed utilizing SwissADME (https://www.swissadme.ch/) and ADMETlab (https://admet.scbdd.com/) (Xiong et al., 2021).

### 2.7 Molecular docking verification

The compound names, molecular weights, and 3D structures of active ingredients were identified from the PubChem database (https://pubchem.ncbi.nlm.nih.gov/), while their corresponding 3D structures were downloaded from the RCSB PDB database (http://www.rcsb.org/) (Berman et al., 2000). Subsequently, ligands and proteins required for molecular docking were prepared using AutoDock software. For target proteins, crystal structures underwent water molecule removal, hydrogen addition, amino acid modification, energy optimization, and force field parameter adjustment to achieve a low-energy conformation for the ligands. Molecular docking was then performed on core targets and compounds with affinity values (in kcal/mol) indicating binding efficiency; lower binding energy indicates more stable ligand-receptor interaction. Finally, Discovery Studio software was utilized to analyze and visualize the docking results by selecting configurations with the best binding energy for each target protein to facilitate further visual analysis.

AutoDock-1.5.6 software (http://vina.scripps.edu/) and Pymol-2.1.0 software were used to prepare ligands and proteins required for molecular docking. For target proteins, The crystal structure obtained in the PDB database (https://www.rcsb.org/) requires pretreatment, including removal of hydrogenation, modification of amino acids, optimization of energy and adjustment of force field parameters. The proteins were hydrogenated and charged with AutoDock Tools-1.5.6 (http://vina.scripps.edu/) and saved as pdbqt format. In the Pubchem database (https://pubchem.ncbi.nlm.nih.gov/) to download ligand structure. Finally will dock target structure and the molecular structure of active ingredients, using pyrx software (https://pyrx.sourceforge.io/) within the docking vina, its Affinity (kcal/mol) value represents the combination of the combination of ability, the lower the combining ability, The more stable the ligand binds to the receptor. Visual analysis of the docking results using Discovery Studio 2019.

### 2.8 Molecular dynamics simulation

Molecular dynamics (MD) simulations are extensively employed in computational studies to investigate the motions of atoms and molecules, playing a pivotal role in assessing protein stability and interactions with docked molecules. The Desmond software was utilized for conducting MD simulations on protein-small molecule complexes (Overton et al., 2021). During the simulation, the OPLS2005 force field was applied to parameterize protein-small molecule interactions, while the TIP3P model was used for simulating water molecules (Li et al., 2021). The protein-small molecule complexes were placed within a cubic water box, and appropriate amounts of chloride and sodium ions were added to neutralize the system’s charge. Prior to simulation, an energy minimization step using the steepest descent method over 50,000 steps ensured a stable initial state. Following equilibration phases, an unrestrained 100 ns simulation was performed to observe dynamic behavior of the protein-small molecule complexes in their free state. To comprehensively document the simulation process, trajectory energy and coordinate data were saved every 10 ps. Throughout the simulation, physiological conditions were emulated by maintaining a temperature of 300 K and pressure at 1 bar.

After conducting molecular dynamics (MD) simulations, the trajectories of the last 10 nanoseconds (ns) were extracted, with a total of 1,000 frames. Then, these frames were used for the calculation of binding free energy using Desmond. Firstly, the MD simulation lasted for a sufficient period of time to ensure that the system reached equilibrium, and the trajectories of the last 10 nanoseconds after equilibrium were extracted. The calculation of each frame was analyzed using the MM-GBSA (Molecular Mechanics Generalized Born Surface Area) module to calculate the binding free energy of the protein-ligand complex. Meanwhile, to gain an in-depth understanding of the contribution of individual amino acids to the binding free energy, energy term decomposition was carried out. The energy terms include: binding free energy (MMGBSA_Bind), Coulomb energy (MMGBSA_Bind_Coulomb), covalent binding energy (MMGBSA_Bind_Covalent), hydrogen bond energy (MMGBSA_Bind_Hbond), hydrophobic energy (MMGBSA_Bind_Lipo), Pi stacking energy (MMGBSA_Bind_Packing), self-contact energy (MMGBSA_Bind_SelfCont), generalized Born solvent effect energy (MMGBSA_Bind_Solv_GB), and van der Waals force energy (MMGBSA_Bind_vdW). In addition, amino acid binding energy decomposition was also performed to analyze the specific contribution of each amino acid to the small molecule binding energy. Through the decomposition of these energy terms, it is possible to have a clearer understanding of each energy term and the influence of each amino acid residue on the overall binding free energy, providing an important basis for further drug design and experimental verification.

The datasets used in this study are sourced from the GEO database (https://www.ncbi.nlm.nih.gov/geo/), with the downloaded data in MINiML format. The detailed processing procedure can be found in the method description on the dataset selection page. Statistical analysis was conducted using R software, version v4.0.3. Results were considered statistically significant when the p-value was less than 0.05.

## 3 Results

### 3.1 Screening the active compounds and putative targets of ZHAST and construction of the “H-C-T” network

**FIGURE 2:**
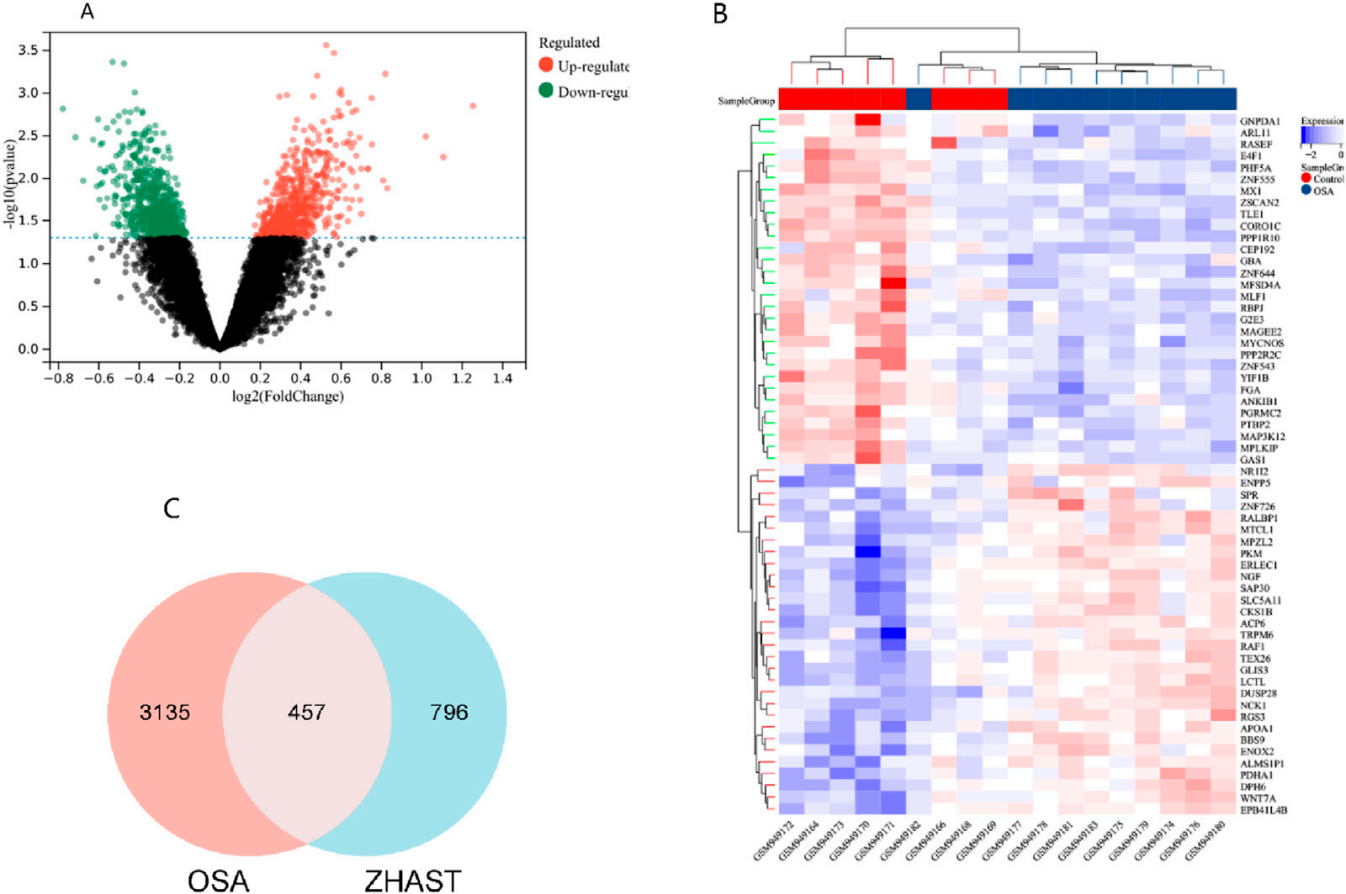
Screening of common targets between ZHAST and OSAHS. **(A)** The volcano plot illustrates the gene distribution in disease samples. Green and red indicate upregulated and downregulated genes, respectively, while grey denotes no significant difference. **(B)** The heatmap depicts the expression patterns of these 60 differentially expressed genes (DEGs). Columns correspond to samples, and rows correspond to genes. **(C)** The Venn diagram shows the 457 common targets between ZHAST active compound targets and OSAHS disease targets.

1. A total of 315 active compounds were isolated from 19 traditional Chinese medicines in the TCMSP database, and after deduplication, 1,252 targets were predicted for these compounds in ZHAST. Integration and deduplication of differentially expressed genes from the GEO database with predicted genes resulted in a total of 3,593 targets analyzed in dataset GSE38792. The differential gene expression is illustrated using a volcano plot (Figure 2A), while Figure 2B shows the heat map of the top 30 differentially expressed genes (DEGs) with color intensities varying according to their log fold changes. Furthermore, OSAHS-related targets were retrieved from the database resulting in identification of 3,135 disease targets after removing redundancies. Cross-analysis between compound targets (796) obtained from ZHAST and disease targets (3,135) identified a total of 457 common targets (Figure 2C).

**FIGURE 3:**
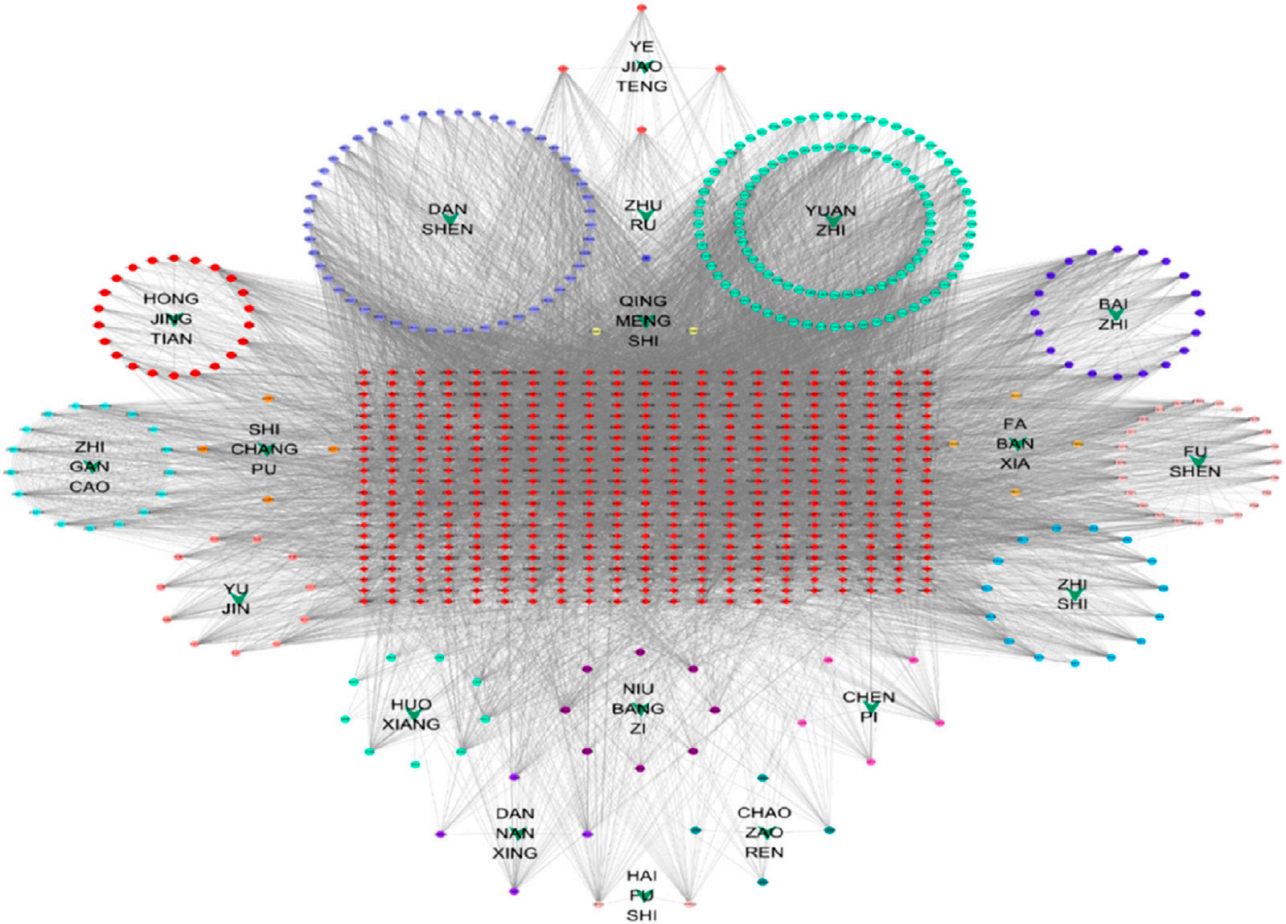
Herb-compound-target network. Green arrows represent the herbs in ZHAST, circular nodes represent compounds, and red squares represent common targets. Edges denote the interactions between compounds and targets.

The H-C-T network is illustrated in Figure 3. Network analysis revealed that HARMINE exhibited the highest degree of connectivity with targets, reaching a value of 91. It was followed by cryptotanshinone with a degree of 70, Thiocyanic Acid with a degree of 65, and both Liquiritigenin and (2R)-7-Hydroxy-2-(4-Hydroxyphenyl)-2,3-Dihydrochromen-4-One with degrees of 63. Additionally, stigmasterol and sesamin displayed equal degrees of connectivity at a value of 23, while Enoxolone had a connectivity degree equal to 58. These results indicate that each protein target is influenced by multiple compounds; conversely, every compound can act on multiple protein targets. Therefore, it is hypothesized that ZHAST may exert its anti-snoring effects in OSAHS through a multi-component and multi-target approach.

### 3.2 PPI network of common targets

**FIGURE 4:**
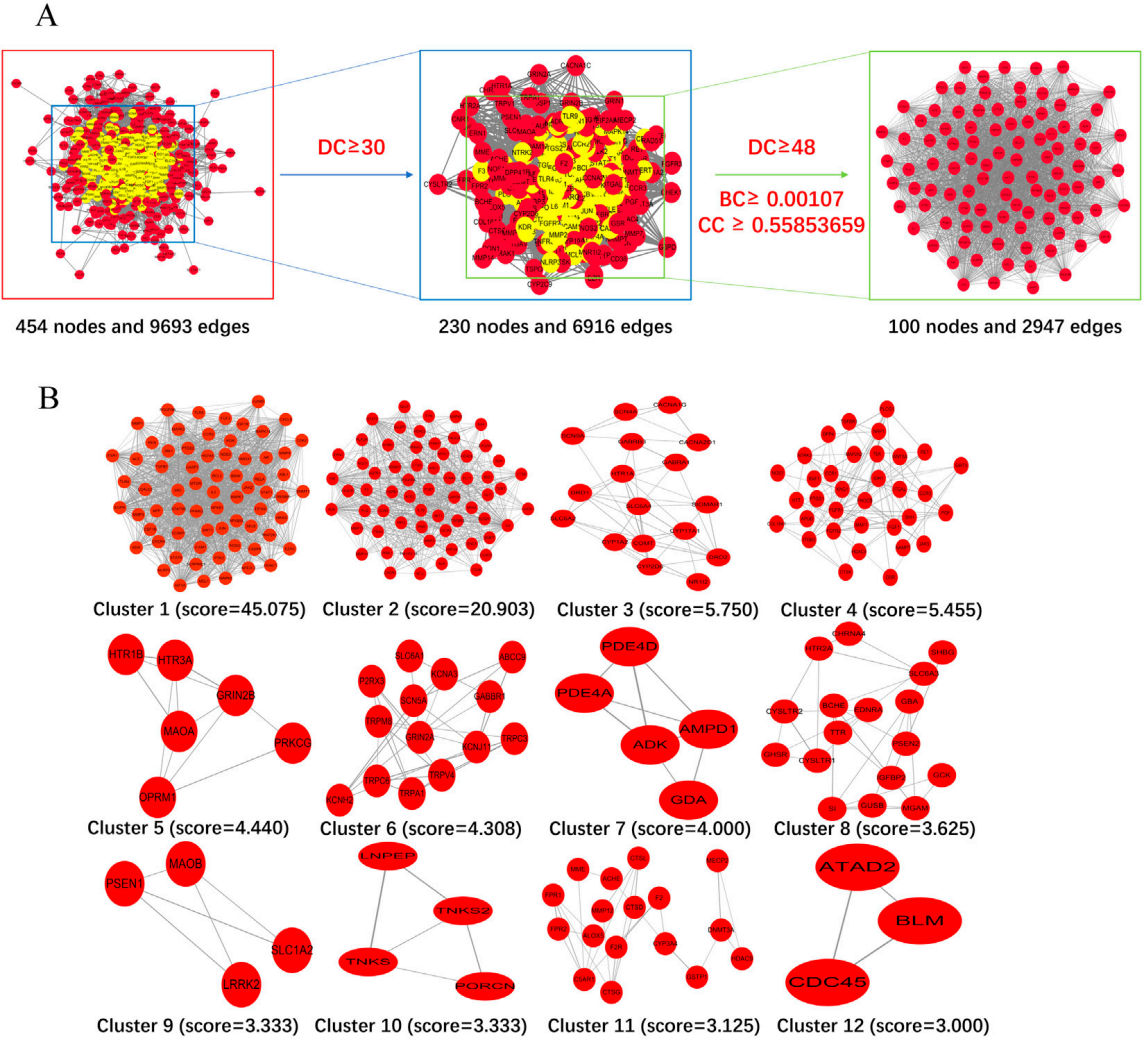
Identification of candidate targets through protein-protein interaction (PPI) analysis. **(A)** Topological filtering process of the PPI network. **(B)** The PPI network based on clustering analysis using the MCODE plugin. **(C)** One hundred core targets selected through degree centrality (DC), betweenness centrality (BC), and closeness centrality (CC), where node size is proportional to the degree of the target in the network. **(D)** Hub genes selected from the PPI network using the CytoHubba plugin. Node colors transition from light yellow to red, corresponding to an increasing degree.

The 457 drug-disease targets were inputted into the STRING database to construct the protein-protein interaction (PPI) network, initially comprising of 454 nodes and 9,693 edges with an average degree of 42.5. To precisely identify central targets, we employed the Cytohubba plugin for Maximum Clique Centrality (MCC) within Cytoscape. Subsequent clustering analysis using the MCODE plugin in Cytoscape revealed the presence of 12 modules within the PPI network. From a pool of one hundred key network targets, ten central targets were further selected as depicted in Figure 4. Based on their degree values, betweenness centrality (BC), and closeness centrality (CC), we determined one hundred core targets (Figures 4A, C). As shown in Figure 4B, clustering analysis using MCODE facilitated the construction of a highly interconnected subnetwork that divided these targets into twelve distinct groups. Utilizing the CytoHubba plugin, we identified the top ten hub genes using MCC method as illustrated in Figure 4D.

### 3.3 Selection and analysis of key targets

**FIGURE 5:**
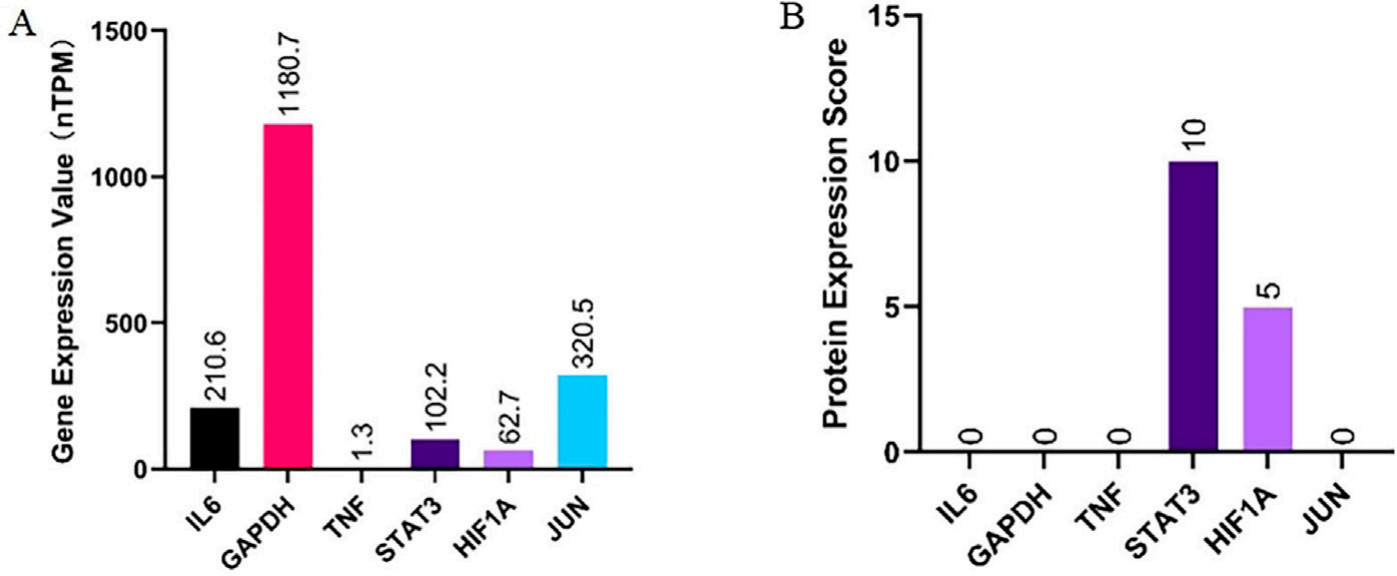
Analysis and summary of the protein expression **(A)** and gene expression **(B)** of key targets in human adipose tissue from the HPA database.

The key targets identified with the highest MCC scores and degree values include TNF, IL6, GAPDH, STAT3, HIF1A, and JUN. To investigate the differential expression of these targets during obstructive sleep apnea progression, we conducted an analysis of protein and gene expression levels in human adipose tissue using the HPA database. Our findings revealed that GAPDH exhibited the highest gene expression levels while TNF displayed the lowest levels. Specifically, JUN had a gene expression level of 320.5 nTPM, STAT3 had 102.2 nTPM, and HIF1A had 62.7 nTPM (Figure 5A). Regarding protein expression patterns in adipose tissue samples, STAT3 and HIF1A demonstrated higher scores compared to IL6, GAPDH, TNF, and JUN which all scored zero (Figure 5B). This suggests that STAT3 and HIF1A may play significant roles in obstructive sleep apnea development with no detectable protein expressions observed for other targets in adipose tissue.

### 3.4 GO enrichment analysis

**FIGURE 6:**
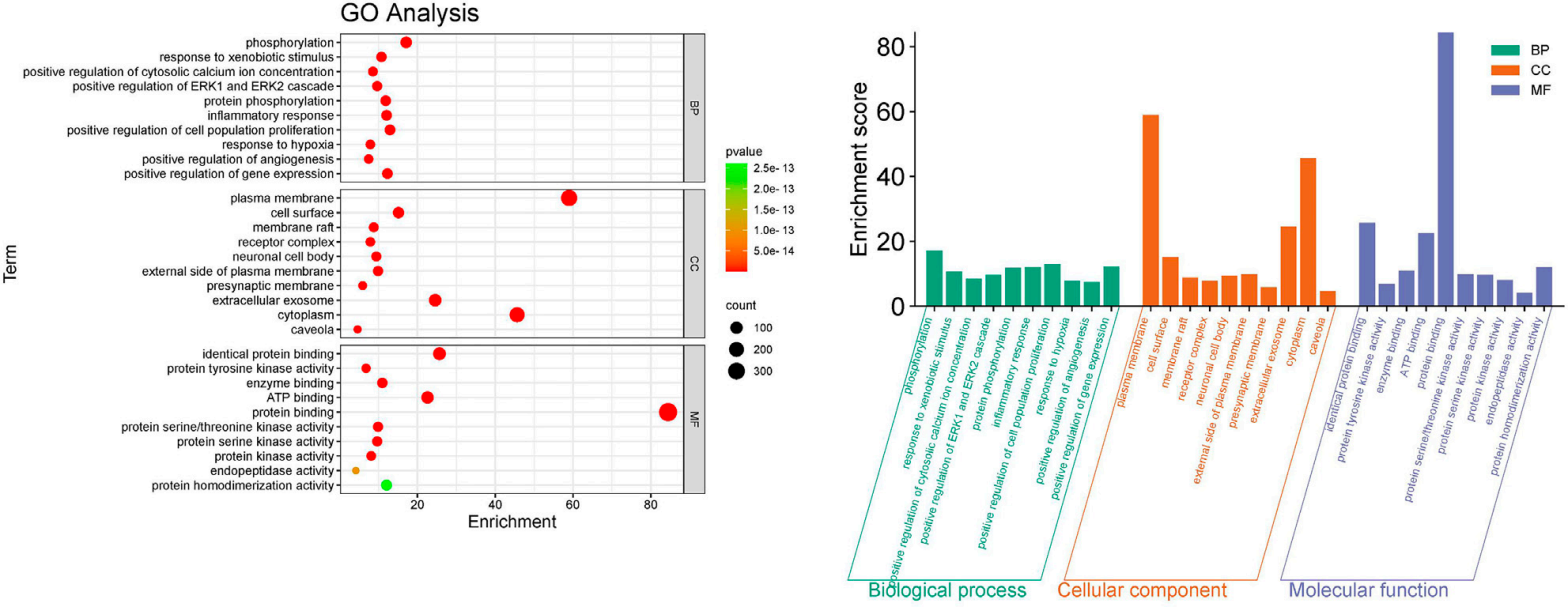
Results of the GO enrichment analysis. **(A)** Bubble chart of the top 10 biological processes (BP), cellular components (CC), and molecular functions (MF). **(B)** The bar chart illustrates the top 10 biological processes (BP), cellular components (CC), and molecular functions (MF), with green, orange, and purple bars representing different categories respectively.

Gene Ontology (GO) enrichment analysis for Biological Processes (BP), Cellular Components (CC), and Molecular Functions (MF) was conducted on 457 potential targets to further explore the mechanisms of ZHAST in treating OSAHS, enriching a total of 1731 GO terms. Among these, 174 were related to CC, 1,261 to BP, and 296 to MF, with the top 10 enriched terms for BP, MF, and CC displayed in Figure 6A. The bar chart in Figure 6B illustrates the terms with the highest gene counts in each category. The analysis reveals that BP terms are primarily associated with cytokine-mediated signaling pathways, inflammatory responses, hypoxia responses, positive regulation of angiogenesis, protein phosphorylation, and regulation of cell proliferation. CC analysis indicates that the relevant components are primarily located on the cell surface, extracellular space, and plasma membrane, including plasma membranes, receptor complexes, and presynaptic membranes. According to MF analysis, several targets are involved in protein binding, protein kinase activity, and protein homodimerization activity.

### 3.5 KEGG analysis

**FIGURE 7:**
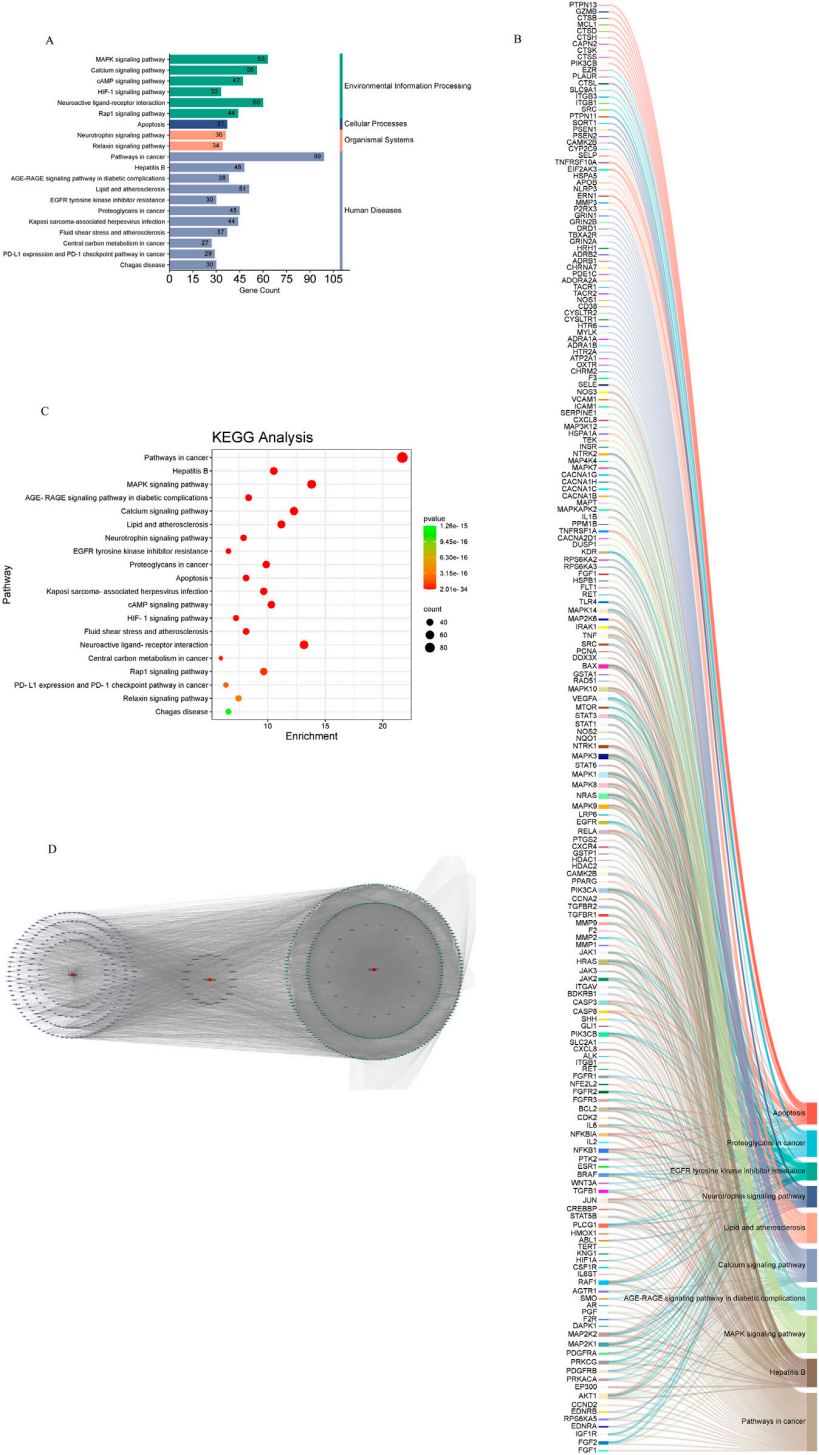
Results of KEGG enrichment analysis and key pathway network construction. **(A)** Bubble chart of the top 20 pathways based on KEGG enrichment analysis. **(B)** Sankey diagram of KEGG pathway analysis for therapeutic targets in the treatment of SONFH with YGPs. In the Sankey diagram, the left rectangular nodes represent therapeutic targets, the right rectangular nodes represent KEGG pathways, and the lines indicate associations between targets and pathways. **(C)** Categories of the top 20 pathways based on KEGG enrichment analysis. **(D)** Composite Target Pathway (C-T-P) network related to the mechanism of action of ZHAST in the treatment of OSAHS. Purple nodes represent targets; pink nodes represent pathways, and green nodes represent compounds.

**FIGURE 8:**
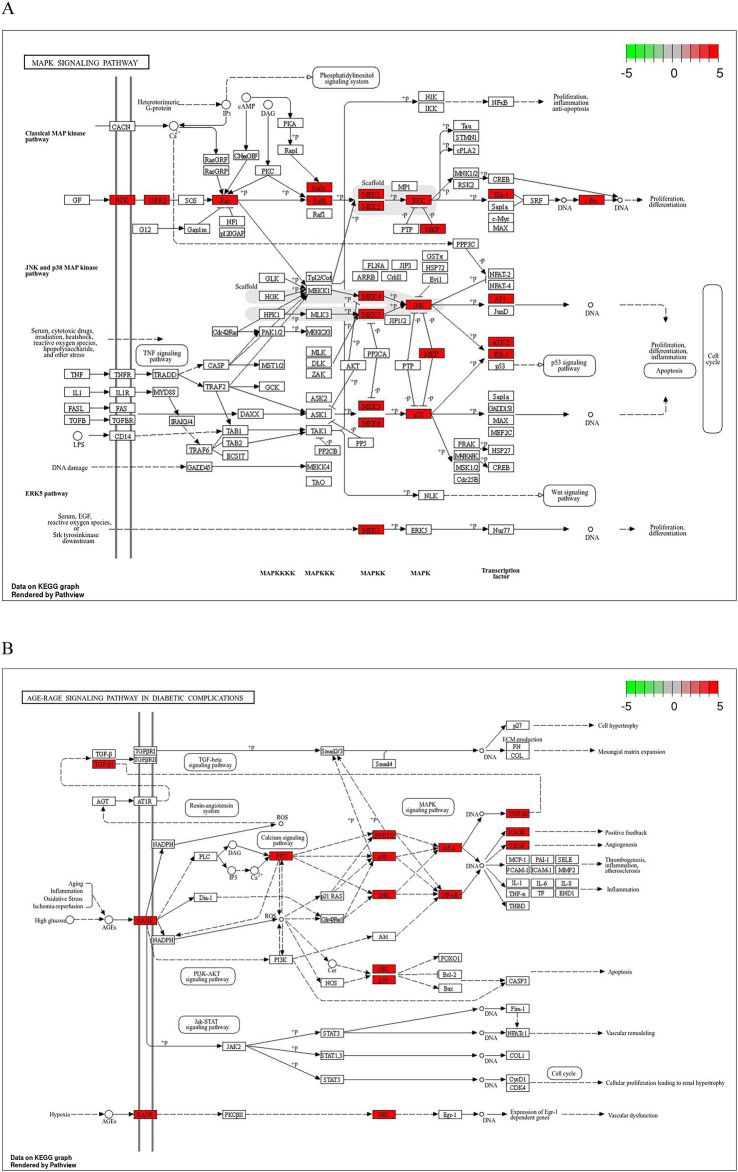
Distribution of key targets in the most relevant pathways. **(A)** Distribution of key targets in the MAPK signaling pathway. **(B)** Distribution of key targets in the AGE-RAGE signaling pathway in diabetic complications. Red rectangles represent key targets, and presumed targets and genes associated with the pathway are indicated in red.

Common drug-disease targets were subjected to KEGG pathway enrichment analysis using DAVID. After filtering for corrected p-values less than 0.05, a total of 203 signaling pathways were identified (Table 1). The top 20 pathways were selected on KEGG enrichment analysis (Figures 7A, C) and were selected to construct the C-T-P network (Figure 7D). A Sankey diagram was created using a free online platform (https://www.bioinformatics.com.cn) to visualize the relationships between enriched pathways and targets (Figure 7B). KEGG pathway enrichment analysis suggests that ZHAST’s pharmacological mechanisms in OSAHS treatment may primarily involve pathways related to cancer, hepatitis B, the MAPK signaling pathway, AGE-RAGE signaling in diabetic complications, calcium signaling, and lipid and atherosclerosis pathways. Furthermore, we visualized the most closely related pathways (MAPK and AGE-RAGE signaling pathway in diabetic complications), with results presented in Figure 8; Table 1.

**TABLE 1:** KEGG enrichment results of key genes.

Pathway ID	Pathway name	Count	Genes	P-value
hsa05200	Pathways in cancer	99	FGF1, FGF2, IGF1R, EDNRA, RPS6KA5, EDNRB, CCND2, AKT1, EP300, PRKACA, PDGFRB, PRKCG, PDGFRA, MAP2K1, MAP2K2, DAPK1, F2R, PGF, AR, SMO, AGTR1, RAF1, IL6ST, CSF1R, HIF1A, KNG1, TERT, ABL1, HMOX1, PLCG1, STAT5B, CREBBP, JUN, TGFB1, WNT3A, BRAF, ESR1, PTK2, NFKB1, IL2, NFKBIA, IL6, CDK2, BCL2, FGFR3, FGFR2, NFE2L2, FGFR1, RET, ITGB1, ALK, CXCL8, SLC2A1, PIK3CB, GLI1, SHH, CASP8, CASP3, BDKRB1, ITGAV, JAK2, JAK3, HRAS, JAK1, MMP1, MMP2, F2, MMP9, TGFBR1, TGFBR2, CCNA2, PIK3CA, PPARG, CAMK2B, HDAC2, HDAC1, GSTP1, CXCR4, PTGS2, RELA, EGFR, LRP6, MAPK9, NRAS, MAPK8, MAPK1, STAT6, MAPK3, NTRK1, NQO1, NOS2, STAT1, STAT3, MTOR, VEGFA, MAPK10, RAD51, GSTA1, BAX	2.01E-34
hsa05161	Hepatitis B	48	DDX3X, PCNA, CXCL8, SRC, PIK3CB, TNF, RELA, MAPK9, NRAS, MAPK8, CASP8, IRAK1, CASP3, AKT1, MAPK1, EP300, STAT6, JAK2, HRAS, JAK3, JAK1, MAPK3, MAP2K6, PRKCG, STAT5B, JUN, MAP2K1, CREBBP, TGFB1, MAP2K2, STAT1, STAT3, BRAF, MAPK14, MMP9, TGFBR1, NFKB1, TGFBR2, MAPK10, NFKBIA, CCNA2, IL6, PIK3CA, CDK2, BCL2, BAX, RAF1, TLR4	3.12E-25
hsa04010	MAPK signaling pathway	63	RET, FLT1, HSPB1, FGF1, FGF2, TNF, IGF1R, RPS6KA3, RPS6KA5, RPS6KA2, CASP3, KDR, AKT1, PRKACA, HRAS, PDGFRB, PRKCG, PDGFRA, MAP2K1, MAP2K2, DUSP1, CACNA2D1, TGFBR1, PGF, TGFBR2, TNFRSF1A, PPM1B, IL1B, MAPKAPK2, MAPT, RAF1, CSF1R, CACNA1B, CACNA1C, EGFR, CACNA1H, RELA, CACNA1G, MAPK9, NRAS, MAPK8, MAPK7, IRAK1, MAPK1, MAPK3, MAP4K4, MAP2K6, NTRK1, NTRK2, JUN, TGFB1, INSR, BRAF, MAPK14, NFKB1, VEGFA, MAPK10, TEK, FGFR3, FGFR2, FGFR1, HSPA1A, MAP3K12	2.03E-24
hsa04933	AGE-RAGE signaling pathway in diabetic complications	38	CXCL8, SERPINE1, PIK3CB, TNF, RELA, ICAM1, MAPK9, NRAS, MAPK8, CASP3, AKT1, MAPK1, PLCG1, JAK2, HRAS, MAPK3, STAT5B, JUN, TGFB1, VCAM1, NOS3, STAT1, MMP2, STAT3, MAPK14, SELE, F3, TGFBR1, NFKB1, TGFBR2, VEGFA, MAPK10, IL6, PIK3CA, IL1B, BCL2, AGTR1, BAX	3.62E-24
hsa04020	Calcium signaling pathway	56	RET, CHRM2, OXTR, FLT1, ATP2A1, HTR2A, ADRA1B, FGF1, ADRA1A, FGF2, MYLK, HTR6, CYSLTR1, EDNRA, CYSLTR2, EDNRB, KDR, CD38, BDKRB1, NOS1, PRKACA, PDGFRB, PRKCG, PDGFRA, F2R, TACR2, TACR1, ADORA2A, AGTR1, CAMK2B, PDE1C, CHRNA7, CACNA1B, CXCR4, ADRB1, CACNA1C, ADRB2, EGFR, CACNA1H, CACNA1G, HRH1, GRIN2A, TBXA2R, DRD1, PLCG1, NTRK1, NTRK2, NOS2, NOS3, GRIN2B, GRIN1, VEGFA, P2RX3, FGFR3, FGFR2, FGFR1	9.77E-23
hsa05417	Lipid and atherosclerosis	51	CXCL8, PIK3CB, TNF, ICAM1, CASP8, CASP3, AKT1, JAK2, HRAS, MMP1, MMP3, MMP9, TNFRSF1A, ERN1, PIK3CA, IL1B, PPARG, TLR4, CAMK2B, SRC, RELA, MAPK9, NRAS, MAPK8, IRAK1, MAPK1, NLRP3, PLCG1, APOB, MAPK3, MAP2K6, JUN, VCAM1, HSPA5, NOS3, STAT3, EIF2AK3, TNFRSF10A, MAPK14, SELE, NFKB1, PTK2, SELP, NFKBIA, MAPK10, CYP2C9, IL6, BCL2, BAX, NFE2L2, HSPA1A	4.35E-22
hsa04722	Neurotrophin signaling pathway	36	CAMK2B, PSEN2, PSEN1, PIK3CB, RELA, MAPK9, RPS6KA3, NRAS, MAPK8, MAPK7, RPS6KA5, IRAK1, RPS6KA2, ABL1, AKT1, MAPK1, PLCG1, HRAS, MAPK3, NTRK1, NTRK2, JUN, MAP2K1, MAP2K2, SORT1, PTPN11, BRAF, MAPK14, NFKB1, MAPK10, NFKBIA, PIK3CA, MAPKAPK2, BCL2, BAX, RAF1	3.29E-19
hsa01521	EGFR tyrosine kinase inhibitor resistance	30	SRC, PIK3CB, FGF2, EGFR, IGF1R, NRAS, KDR, AKT1, MAPK1, PLCG1, JAK2, HRAS, JAK1, MAPK3, PRKCG, PDGFRB, PDGFRA, MAP2K1, MAP2K2, STAT3, BRAF, MTOR, VEGFA, IL6, PIK3CA, BCL2, BAX, RAF1, FGFR3, FGFR2	4.98E-19
hsa05205	Proteoglycans in cancer	45	ITGB1, CAMK2B, SRC, ITGB3, PIK3CB, FGF2, HIF1A, TNF, EGFR, SLC9A1, IGF1R, SHH, NRAS, CTSL, CASP3, KDR, AKT1, MAPK1, ITGAV, PLCG1, PRKACA, HRAS, MAPK3, PRKCG, MAP2K1, TGFB1, MAP2K2, WNT3A, MMP2, STAT3, PLAUR, PTPN11, BRAF, MAPK14, MMP9, ESR1, MTOR, PTK2, VEGFA, SMO, PIK3CA, EZR, RAF1, TLR4, FGFR1	2.82E-18
hsa04210	Apoptosis	37	PIK3CB, TNF, RELA, CTSS, MAPK9, NRAS, MAPK8, CASP8, CTSL, CASP3, CTSK, CAPN2, AKT1, MAPK1, CTSH, HRAS, CTSD, MCL1, MAPK3, CTSB, NTRK1, JUN, MAP2K1, MAP2K2, EIF2AK3, GZMB, TNFRSF10A, PTPN13, NFKB1, TNFRSF1A, ERN1, MAPK10, NFKBIA, PIK3CA, BCL2, BAX, RAF1	3.37E-18
hsa05167	Kaposi sarcoma-associated herpesvirus infection	44	CXCL8, SRC, PIK3CB, PTGS2, FGF2, HIF1A, RELA, ICAM1, MAPK9, NRAS, MAPK8, CASP8, CASP3, AKT1, MAPK1, EP300, PLCG1, JAK2, CCR5, HRAS, CCR3, JAK1, MAPK3, MAP2K6, CCR1, JUN, MAP2K1, CREBBP, MAP2K2, STAT1, STAT3, MAPK14, MTOR, NFKB1, TNFRSF1A, VEGFA, MAPK10, NFKBIA, IL6, PIK3CA, MAPKAPK2, BAX, RAF1, IL6ST	3.55E-18
hsa04024	cAMP signaling pathway	47	CHRM2, CAMK2B, GLP1R, OXTR, ADRB1, ATP2A1, CACNA1C, ADRB2, ATP1A1, PIK3CB, GLI1, RELA, SLC9A1, MAPK9, GRIN2A, MAPK8, HTR6, EDNRA, ADORA1, AKT1, PDE4A, MAPK1, EP300, DRD1, DRD2, PRKACA, MAPK3, GHSR, JUN, MAP2K1, CREBBP, GABBR1, MAP2K2, PDE4D, F2R, HTR1A, HTR1B, BRAF, GRIN2B, NFKB1, GRIN1, MAPK10, NFKBIA, ADORA2A, PIK3CA, RAF1, CFTR	5.23E-18
hsa04066	HIF-1 signaling pathway	33	CAMK2B, FLT1, SERPINE1, SLC2A1, PIK3CB, HIF1A, RELA, EGFR, IGF1R, HK1, AKT1, HMOX1, MAPK1, EP300, PLCG1, MAPK3, PRKCG, MAP2K1, CREBBP, MAP2K2, NOS2, NOS3, INSR, STAT3, MTOR, NFKB1, VEGFA, IL6, PIK3CA, BCL2, TEK, GAPDH, TLR4	9.42E-18
hsa05418	Fluid shear stress and atherosclerosis	37	SRC, GSTP1, ITGB3, PIK3CB, TNF, RELA, ICAM1, MAPK9, MAPK8, MAPK7, CTSL, KDR, AKT1, HMOX1, ITGAV, MAP2K6, NQO1, JUN, VCAM1, NOS3, DUSP1, MMP2, MAPK14, SELE, MMP9, PTK2, NFKB1, TNFRSF1A, VEGFA, MAPK10, PIK3CA, TRPV4, IL1B, GSTA1, BCL2, BMPR1A, NFE2L2	1.21E-17
hsa04080	Neuroactive ligand-receptor interaction	60	GABRB3, GLP1R, CHRM2, OXTR, HTR2A, GRIK2, ADRA1B, NR3C1, ADRA1A, RXFP1, LTB4R, MC4R, HTR6, CYSLTR1, EDNRA, CYSLTR2, EDNRB, ADORA3, ADORA1, GNRHR, TSPO, BDKRB1, CTSG, GHSR, AVPR2, F2R, TACR2, TACR1, OPRM1, F2, ADRA2A, SSTR4, ADORA2A, AGTR1, CHRNA1, CHRNA4, CHRNA7, C5AR1, FPR1, ADRB1, PLG, ADRB2, FPR2, KNG1, HRH1, GRIN2A, TBXA2R, CNR1, DRD1, DRD2, GABRA1, GABBR1, GABRA5, HTR1A, HTR1B, TRPV1, GRIN2B, HCRTR2, GRIN1, P2RX3	1.34E-17
hsa05230	Central carbon metabolism in cancer	27	RET, SLC2A1, PIK3CB, HIF1A, EGFR, HK1, NRAS, AKT1, MAPK1, HRAS, MAPK3, PDGFRB, NTRK1, PDGFRA, MAP2K1, G6PD, MAP2K2, IDH1, GCK, MTOR, SIRT3, PKM, PIK3CA, RAF1, FGFR3, FGFR2, FGFR1	2.60E-17
hsa04015	Rap1 signaling pathway	44	ITGB1, CSF1R, FLT1, SRC, ITGB3, FPR1, PIK3CB, FGF1, ITGAL, FGF2, EGFR, IGF1R, GRIN2A, NRAS, CNR1, KDR, AKT1, MAPK1, PLCG1, DRD2, HRAS, MAPK3, MAP2K6, PRKCG, PDGFRB, PDGFRA, MAP2K1, MAP2K2, INSR, F2R, BRAF, MAPK14, GRIN2B, PGF, GRIN1, VEGFA, ADORA2A, PIK3CA, PRKD1, TEK, RAF1, FGFR3, FGFR2, FGFR1	7.74E-17
hsa05235	PD-L1 expression and PD-1 checkpoint pathway in cancer	29	ALK, PIK3CB, HIF1A, RELA, EGFR, NRAS, AKT1, MAPK1, PLCG1, JAK2, HRAS, JAK1, MAPK3, MAP2K6, JUN, MAP2K1, MAP2K2, STAT1, STAT3, PTPN11, MAPK14, MTOR, NFKB1, NFKBIA, PIK3CA, CSNK2B, TLR9, RAF1, TLR4	2.06E-16
hsa04926	Relaxin signaling pathway	34	SRC, PIK3CB, RXFP1, RELA, EGFR, MAPK9, NRAS, MAPK8, EDNRB, AKT1, MAPK1, NOS1, PRKACA, HRAS, MAPK3, JUN, MAP2K1, TGFB1, MAP2K2, NOS2, MMP1, NOS3, MMP2, MAPK14, MMP9, TGFBR1, NFKB1, TGFBR2, VEGFA, MAPK10, NFKBIA, MMP13, PIK3CA, RAF1	3.45E-16
hsa05142	Chagas disease	30	CXCL8, SERPINE1, PIK3CB, TNF, RELA, KNG1, MAPK9, MAPK8, CASP8, IRAK1, AKT1, MAPK1, MAPK3, JUN, TGFB1, ACE, NOS2, MAPK14, TGFBR1, IL2, NFKB1, TGFBR2, TNFRSF1A, MAPK10, NFKBIA, IL6, PIK3CA, IL1B, TLR9, TLR4	1.26E-15

### 3.6 ADME and toxicity analysis

The absorption, distribution, metabolism, excretion, and toxicity (ADMET) properties of ten selected drugs were assessed using SwissADME (https://www.swissadme.ch/) and ADMETlab (https://admet.scbdd.com/). The results are presented in Tables 2, 3.

**TABLE 2:** Presents the pharmacokinetic parameters.

	5-[(2S,3S)-7-methoxy-3-methyl-5-[(E)-prop-1-enyl]-2,3-dihydrobenzofuran-2-yl]-1,3-benzodioxole	meso-1,4-Bis-(4-hydroxy-3-methoxyphenyl)-2,3-dimethylbutane	threo-austrobailignan-5	Isoguaiacin	Kudos	Galbacin
GI absorption	High	High	High	High	High	High
BBB permeant	Yes	Yes	No	Yes	Yes	No
P-gp substrate	No	Yes	No	Yes	Yes	Yes
CYP1A2 inhibitor	Yes	Yes	No	Yes	Yes	No
CYP2C19 inhibitor	No	Yes	No	No	No	No
CYP2C9 inhibitor	No	Yes	No	No	No	No
CYP2D6 inhibitor	Yes	No	No	No	No	No
CYP3A4 inhibitor	Yes	Yes	No	No	No	No
Log Kp (skin permeation)	−4.94 cm/s	−5.41 cm/s	−6.26 cm/s	−6.23 cm/s	−6.23 cm/s	−5.27 cm/s

**TABLE 3:** Displays the toxicological parameters.

	5-[(2S,3S)-7-methoxy-3-methyl-5-[(E)-prop-1-enyl]-2,3-dihydrobenzofuran-2-yl]-1,3-benzodioxole	meso-1,4-Bis-(4-hydroxy-3-methoxyphenyl)-2,3-dimethylbutane	threo-austrobailignan-5	Isoguaiacin	Kudos	Galbacin
hERG Blockers	0.396	0.058	0.001	0.248	0.248	0.045
hERG Blockers (10um)	0.566	0.418	0.175	0.624	0.624	0.241
DILI	0.918	0.896	0.993	0.277	0.277	0.079
AMES Toxicity	0.853	0.889	0.195	0.615	0.615	0.052
Rat Oral Acute Toxicity	0.723	0.576	0.989	0.487	0.487	0.247
FDAMDD	0.705	0.846	0.028	0.636	0.636	0.787
Skin Sensitization	0.273	0.992	1	0.619	0.619	0.211
Carcinogenicity	0.87	0.968	0.163	0.719	0.719	0.21
Eye Corrosion	0.002	0.003	1	0.066	0.066	0.004
Eye Irritation	0.944	0.682	1	0.991	0.991	0.242
Respiratory	0.937	0.497	1	0.499	0.499	0.841
Human Hepatotoxicity	0.681	0.865	0.964	0.691	0.691	0.437
Drug-induced Nephrotoxicity	0.874	0.963	0.991	0.271	0.271	0.078
Drug-induced Neurotoxicity	0.85	0.754	0.318	0.611	0.611	0.271
Ototoxicity	0.61	0.616	0.029	0.271	0.271	0.896
Hematotoxicity	0.606	0.918	0.83	0.137	0.137	0.153
Genotoxicity	0.494	0.975	0.996	0.779	0.779	0.029
RPMI-8226 Immunitoxicity	0.149	0.202	0.02	0.044	0.044	0.025
A549 Cytotoxicity	0.313	0.195	0	0.222	0.222	0.089
Hek293 Cytotoxicity	0.456	0.761	0.002	0.8	0.8	0.17
BCF	1.122	2.104	0.893	1.246	1.246	1.187
IGC50	3.558	5.307	3.169	3.644	3.644	4.248
LC50DM	4.434	6.898	5.744	4.331	4.331	5.868
LC50FM	4.062	6.614	3.955	4.024	4.024	4.897

### 3.7 Molecular docking

**FIGURE 9:**
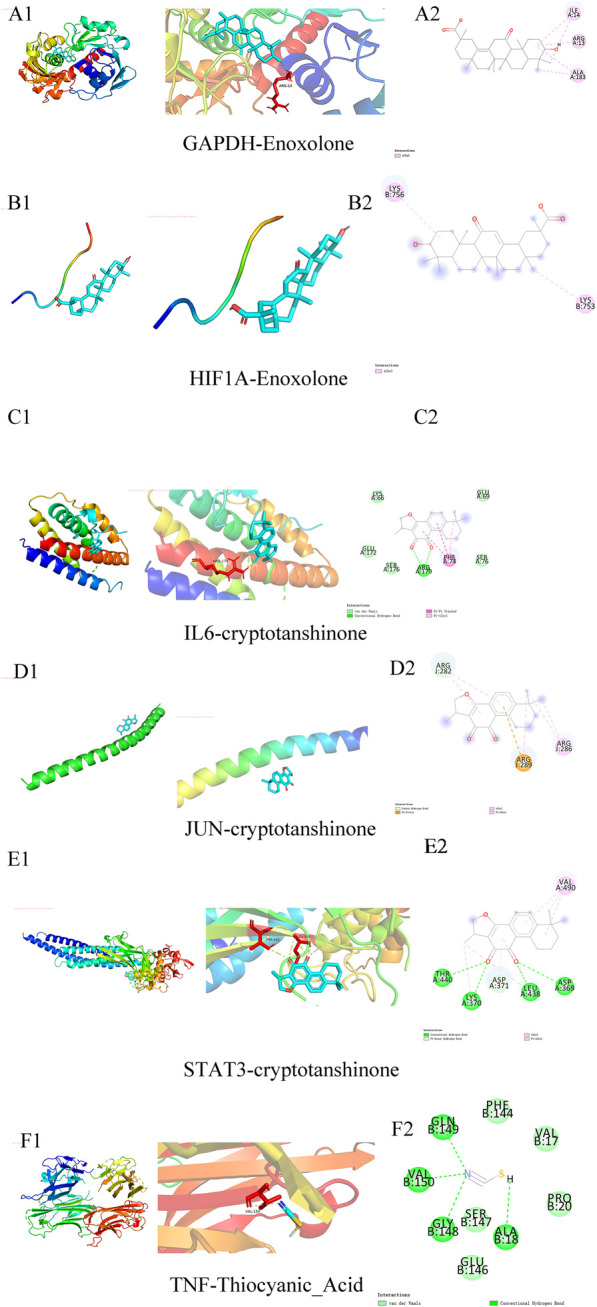
Docking patterns of key targets with specific active compounds.

**FIGURE 10:**
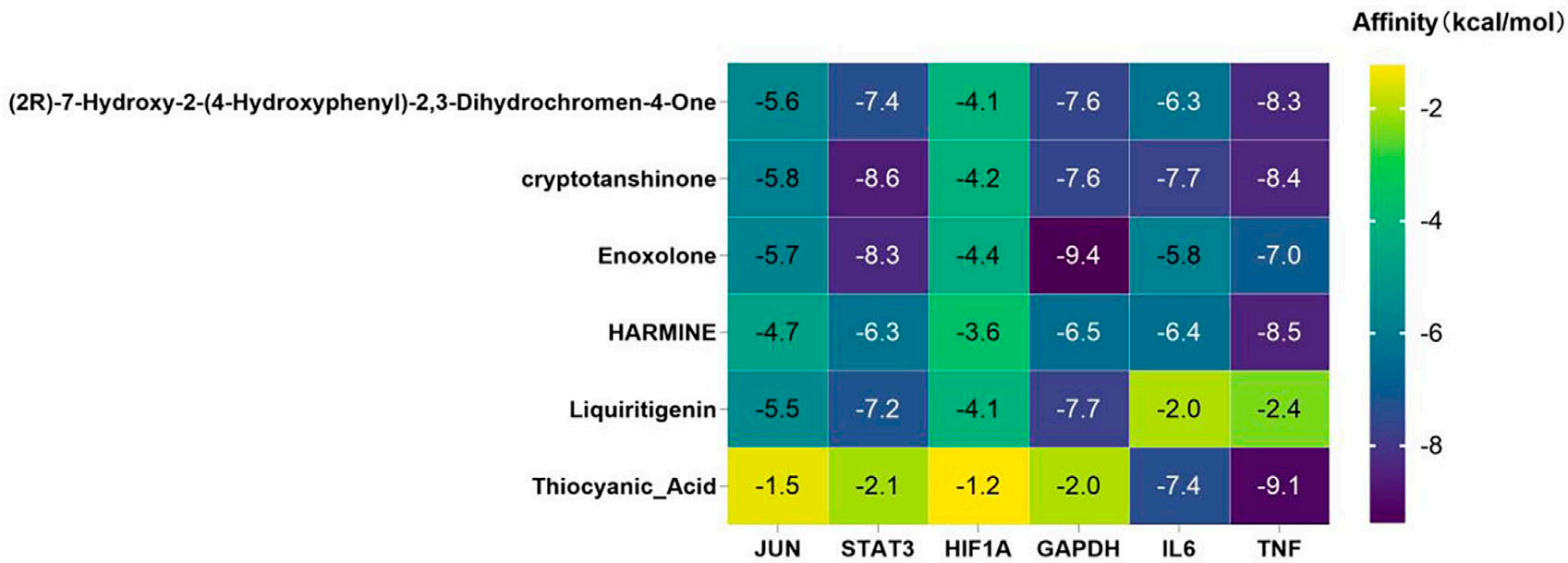
Binding affinities (kcal/mol) of key targets and active compounds in traditional Chinese medicine.

Molecular docking was performed between six core targets and six core compounds, where the Affinity (kcal/mol) values represent the binding strength. Lower values indicate more stable ligand-receptor interactions, as shown in Figure 10. Finally, the Discovery Studio software was used to analyze and visualize the docking results. The binding energies with the best scores for each target protein were plotted, as illustrated in Figures 9, 10. Observed binding energies from the docking ranged between −1.2 and −9.4 kcal/mol. Results indicated that among the six key targets, GAPDH exhibited the lowest binding energy with the compounds. Of the five ligands docked with GAPDH, Enoxolone demonstrated the lowest energy, at −9.4 kcal/mol. Enoxolone was able to form a hydrogen bond with the active pocket amino acid residue ARG13, as shown in Figures 9A1, 9A2. In studies involving HIF1A, Enoxolone’s binding energy was notably low at −4.4 kcal/mol, primarily interacting through hydrogen bonds with residues LYSB753 and LYSB756, as illustrated in Figures 9B1, 9B2.Additionally, the binding site of IL6 demonstrated affinity for cryptotanshinone, with a binding energy of −7.7 kcal/mol, predominantly achieved through hydrogen bonding with the PHEA74 residue, as depicted in Figures 9C1, 9C2. In molecular docking analyses, the interaction between JUN and cryptotanshinone showed substantial affinity, with the lowest energy recorded at −5.8 kcal/mol, facilitated by a hydrogen bond at ARGJ286, as illustrated in Figures 9D1, 9D2.The interaction between STAT3 and cryptotanshinone exhibited a minimal binding energy of −8.6 kcal/mol, primarily supported by hydrogen bonding at the VALA490 residue (Figures 9E1, 9E2). Similarly, the binding affinity between TNF and Thiocyanic Acid was notably high, at −9.1 kcal/mol, largely due to a hydrogen bond involving the ALAB18 residue (Figures 9F1, 9F2). Notably, cryptotanshinone and Enoxolone demonstrated significant binding affinity with target proteins, suggesting that these compounds could play a critical role in the therapeutic effects of ZHAST.

### 3.8 Molecular dynamics simulation

**FIGURE 11:**
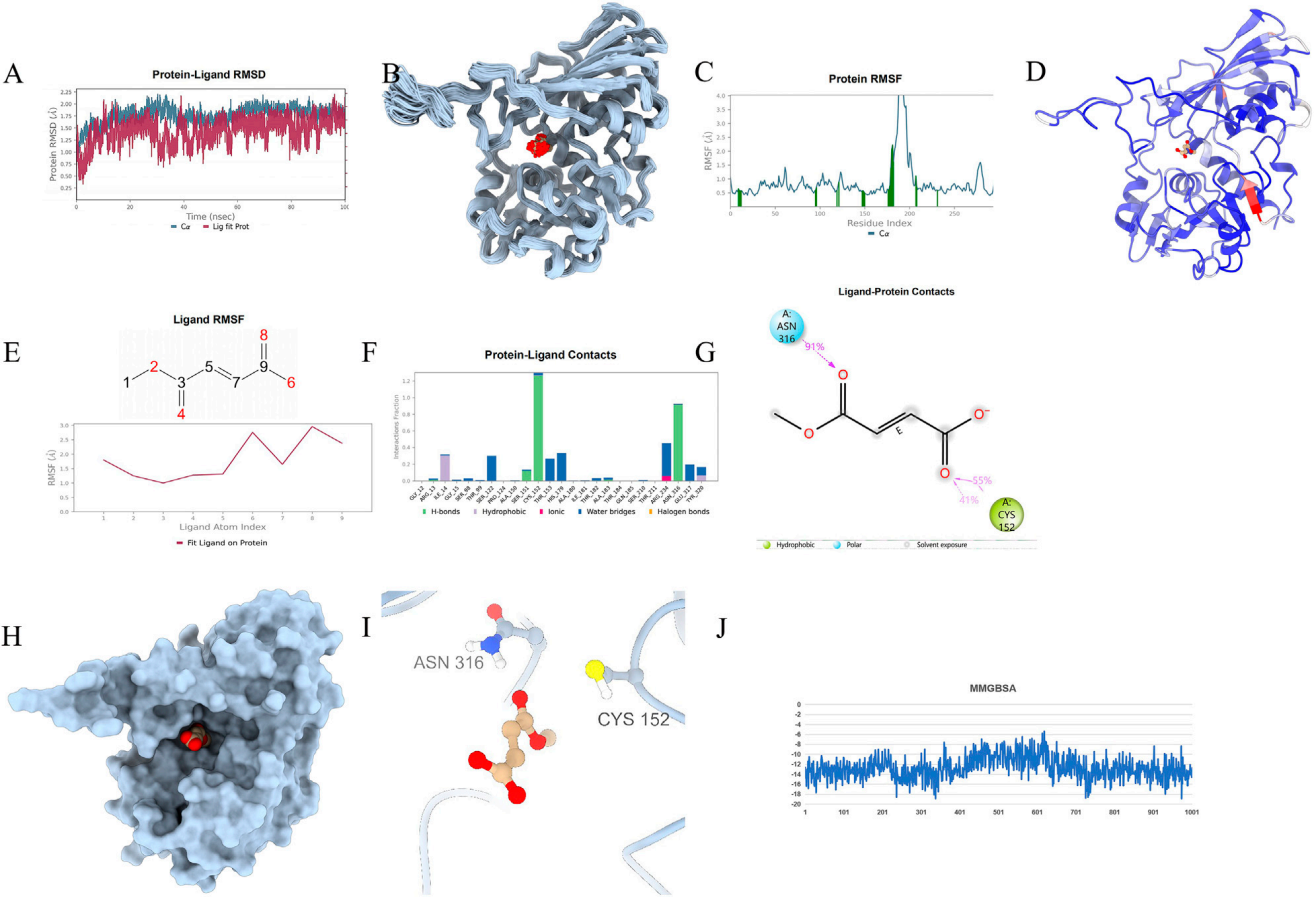
Molecular Simulation Results of protein-proligand complex. **(A)** RMSD; **(B)**: Overlay of 100 conformations sampled every 1 ns during the 100 ns molecular dynamics simulation. **(C)**: RMSF analysis of the protein during the 40–100 ns segment of the molecular dynamics simulation, with interacting amino acid positions marked in green. **(D)**: B-factor distribution calculated from the MD simulation trajectory, indicating regions of high flexibility in red and low flexibility in blue; **(E)** Ligand RMSF; **(F, G)** The connection between ligand and protein. **(H, I)** The three-dimensional schematic diagram of the binding mode of small molecules and protein after molecular dynamics optimization; **(J)** MMGBSA.

**FIGURE 12:**
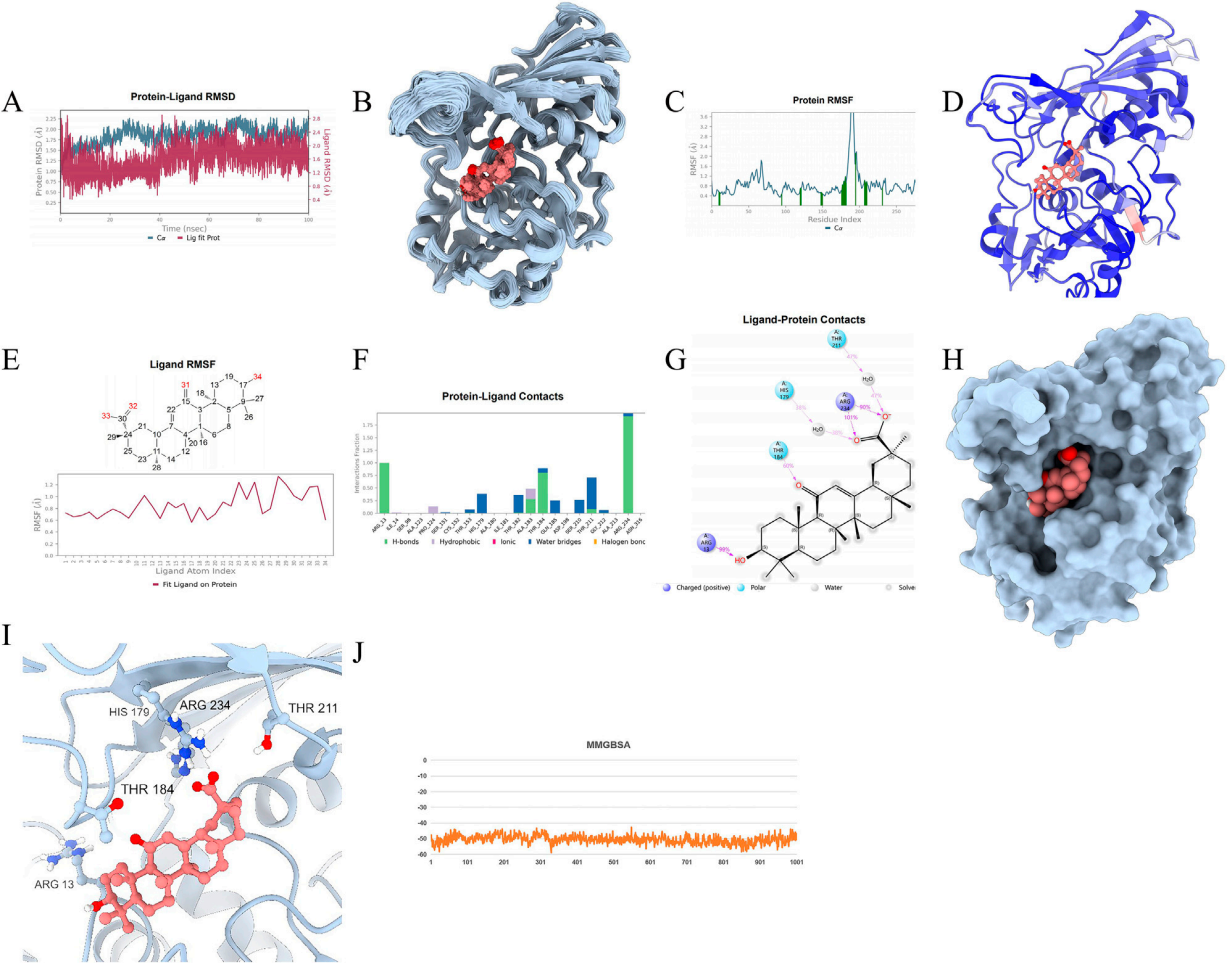
Molecular Simulation Results of protein-drug molecular complex. **(A)** RMSD; **(B)**: Overlay of 100 conformations sampled every 1 ns during the 100 ns molecular dynamics simulation. **(C)**: RMSF analysis of the protein during the 40–100 ns segment of the molecular dynamics simulation, with interacting amino acid positions marked in green. **(D)**: B-factor distribution calculated from the MD simulation trajectory, indicating regions of high flexibility in red and low flexibility in blue; **(E)** Ligand RMSF; **(F, G)** The connection between ligand and protein. **(H, I)** The three-dimensional schematic diagram of the binding mode of small molecules and protein after molecular dynamics optimization; **(J)** MMGBSA.

**FIGURE 13:**
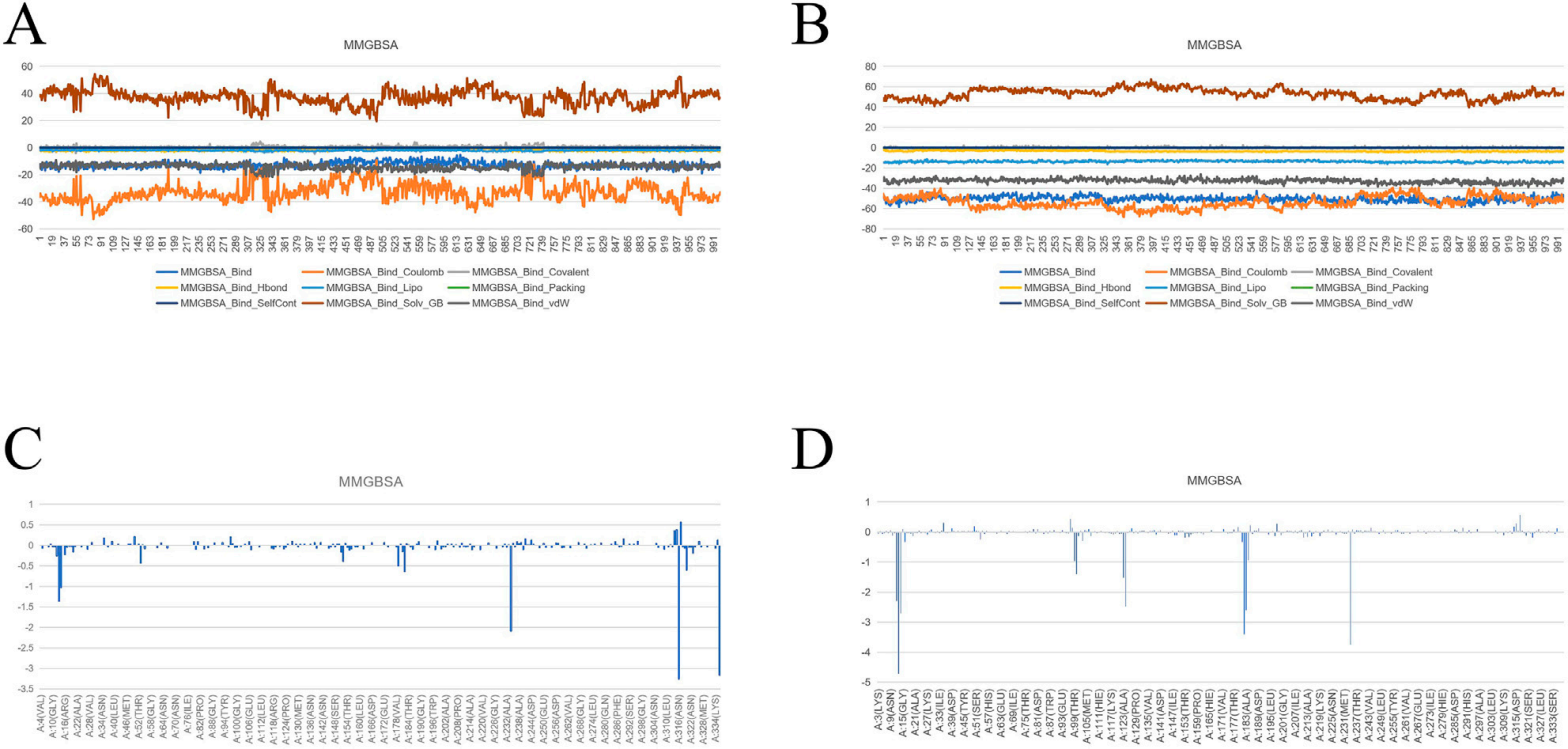
**(A)** MMGBSA of protein-proligand complex. **(B)** MMGBSA of protein-drug molecular complex. **(C)** The binding free energy of the original ligand and the protein is decomposed to the energy of each amino acid residue. **(D)** The binding free energy of the drug and the protein is decomposed to the energy of each amino acid residue.

**FIGURE 14:**
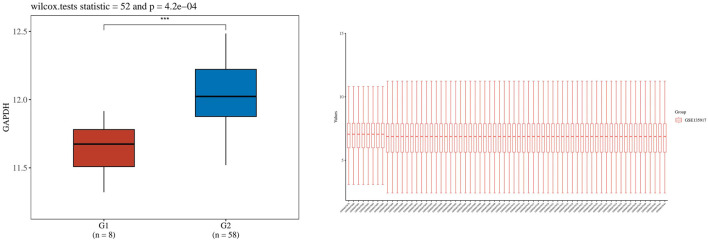
Shows the boxplot of GADPH gene expression distribution in lung tissue and normal tissue. The horizontal coordinate represents the different sample groups, and the vertical coordinate represents the distribution of the expression of the gene. Different colors represent different groups. The asterisk in the upper left represents the significance P-value, where * means that the P-value is less than 0.05, ** means that the P-value is less than 0.01, *** means that the P-value is less than 0.001, and the number of asterisks represents the degree of significance.

To ascertain the stability of the protein-ligand complex, protein-drug molecular complex, molecular dynamics (MD) simulations were conducted. A 100 ns MD simulation was performed on the GAPDH-Enoxolone complex, analyzing molecular mobility, trajectories, and conformational changes based on the docking data. Initially, the Root Mean Square Deviation (RMSD) data for GAPDH and Enoxolone were extracted during the simulation. As depicted in Figures 11A, 12A, the RMSD curves indicate that following 40 ns, the RMSD values for GAPDH and Enoxolone stabilized, suggesting that the protein-ligand complex achieved a relatively stable state. Based on this observation, the simulation trajectories from 40 to 100 ns were selected for further sampling analysis.Furthermore, a conformation was saved every 1 ns during the simulation, totaling 100 conformations, which were subsequently superimposed. As shown in Figures 11B, 12B, these superimposed conformations demonstrated good consistency, indicating that the conformations of GAPDH protein and the Enoxolone molecule maintained high stability throughout the simulation. Concurrently, the small molecule was able to stably bind to the protein’s active site. Upon detailed analysis of the simulation trajectory, the Root Mean Square Fluctuation (RMSF) data for GAPDH protein and the Enoxolone molecule were extracted, and the corresponding B-factors were calculated (as illustrated in Figures 11C, 12C). From the RMSF and B-factor graphs, it is evident that the overall structure of the GAPDH protein exhibits low flexibility (with RMSF values below 2.0 Å for most regions), indicating that the protein structure maintained high stability throughout the simulation. Notably, the RMSF values near the binding site of the Enoxolone molecule are also low, further corroborating the exceptional stability of the region where the molecule binds during the simulation. Compared with RMSF of the protein in the protein - native ligand complex, the RMSF of the protein in the protein - drug molecule complex is lower, indicating that the protein structure is more stable (as illustrated in Figures 13, 14).

## 4 Discussion

Obstructive sleep apnea-hypopnea syndrome (OSAHS) is a prevalent sleep disorder characterized by recurrent upper airway obstructions and apneas during sleep (Schweitzer et al., 2023). Untreated OSAHS patients are at an elevated risk of developing cardiovascular diseases, diabetes, and other health complications (Chen et al., 2016). Current treatment options encompass Continuous Positive Airway Pressure (CPAP) therapy and lifestyle modifications; however, their suitability varies among patients. Therefore, it is crucial to explore additional therapeutic alternatives (Wang N. et al., 2024).

“The traditional Chinese medicine formula ZhihanAnShenTang, developed based on the principles of traditional Chinese medicine, holds potential for individuals suffering from OSAHS. However, a comprehensive understanding of its bioactive components and their mechanisms of action is still lacking. Therefore, our study aims to employ network pharmacology and molecular docking strategies to investigate the potential compounds in ZHAST and elucidate their mechanisms of action in treating OSAHS.”

Utilizing the TCMSP database, we conducted a screening of 315 active compounds in ZHAST and employed the Swiss Target Prediction web server to predict 1,252 targets. Among these targets, harmine, cryptotanshinone, thiocyanic acid, liquiritigenin, (2R)-7-hydroxy-2-(4-hydroxyphenyl)-2,3-dihydrochromen-4-one, and enoxolone were identified as active compounds associated with a majority of targets. Previous studies have demonstrated the efficacy of these compounds in treating OSAHS. Helenalin is a naturally occurring sesquiterpene lactone that exhibits diverse pharmacological activities including antitumor, antiviral, and neuroprotective effects (Zhao et al., 2021). Recent investigations have indicated that harmine effectively inhibits the migration and viability of MRC-5 cells induced by TGF-β1 (Gong et al., 2024). This inhibition is achieved through apoptosis induction in these cells and suppression of F-actin expression which prevents phenotypic transformation of pulmonary fibroblasts into myofibroblasts - a crucial process for pulmonary disease progression. According to Li et al., CPT functions by modulating gut microbiota and bile acid metabolism, specifically inhibiting epithelial-mesenchymal transition (EMT) and inflammatory responses, thereby reducing the deposition of extracellular matrix during pulmonary injury (Li et al., 2023). Liquiritigenin, a flavanone compound extracted from licorice (Glycyrrhiza), is renowned for its diverse biological activities, including anti-inflammatory and anti-cancer properties (Liu et al., 2024; Wang et al., 2012b). This compound modulates various cellular signaling pathways that impact cell proliferation, migration, and apoptosis. Studies have demonstrated that liquiritigenin inhibits the migration of human lung adenocarcinoma A549 cells by downregulating proMMP-2 expression and suppressing the activation of the PI3K/Akt signaling pathway (Wang et al., 2012b). Its significant anti-inflammatory and anti-cancer properties were highlighted in Luo’s study where it exhibited efficacy in inducing apoptosis, causing G2/M cell cycle arrest, and inhibiting migration in A549 lung cancer cells through modulation of various signaling pathways (Luo et al., 2021). 18β-Glycyrrhetinic acid significantly impacts intracellular mechanisms such as increasing levels of reactive oxygen species (ROS) and regulating signaling pathways like MAPK, STAT3, and NF-κB which are crucial for managing the proliferation and metastasis of lung cancer cells (Ji et al., 2021). Therefore, ZHAST’s multiple active compounds can effectively treat OSAHS through diverse mechanisms.IL-6 is a multifunctional cytokine that is commonly associated with inflammation and immune responses, produced by various cell types, and plays a crucial role in autoimmune diseases and chronic inflammatory conditions. IL-6 serves as a bridge between infections, stress responses, and both acute and chronic inflammation. In pulmonary diseases, particularly in inflammation associated with OSAHS, IL-6 promotes inflammatory and tissue damage responses (Fiedorczuk et al., 2023). IL-6 intensifies the progression of pulmonary diseases by activating immune cells in the lungs and promoting the release of inflammatory cytokines. Under pulmonary pathological conditions, particularly in cancer or inflammatory diseases, GAPDH’s role extends beyond its metabolic functions (Fu et al., 2023). Studies indicate that GAPDH may regulate cell proliferation and apoptosis, which are key processes in cancer development and progression. For instance, existing research demonstrates that GAPDH exhibits apoptotic and antiproliferative potential against lung cancer cells in both *in vitro* and *in vivo* models (Sakthidhasan et al., 2022). Within the context of pulmonary cells, STAT3 is implicated in responding to environmental stressors and inflammatory signals prevalent in respiratory diseases. Activation of STAT3 can influence the expression of multiple genes involved in inflammatory responses, cell proliferation, and survival—critical processes for the pathogenesis of lung diseases (Wang et al., 2023). In OSAHS (obstructive sleep apnea-hypopnea syndrome), intermittent hypoxia—a hallmark of this condition—can activate pathways including STAT3, playing a role in cellular response to hypoxic stress. Furthermore, Bao (2020) discussed the role of STAT3 mediating the effects of intermittent hypoxia on cardiac fibrosis, highlighting variable impacts from STAT3 activation across different tissue types. HIF-1α is a transcription factor that plays a central role in cellular responses to hypoxic environments (Bao et al., 2020). Comprising two subunits (HIF-1α and HIF-1β), HIF-1α is degraded under normoxic conditions but stabilizes and activates various gene expressions under hypoxic conditions. These genes are involved with regulating erythropoiesis, energy metabolism, and cell survival (Mesarwi et al., 2021). The activity of HIF-1 within pulmonary cells is crucial for lung health—particularly regulating oxygenation status—and for managing pulmonary diseases such as fibrosis inflammation angiogenesis through gene activation (Lin et al., 2020).

Functional analysis based on Gene Ontology (GO) reveals that cytokine-mediated signaling pathways, inflammatory responses, hypoxic responses, positive regulation of angiogenesis, protein phosphorylation, and regulation of cell proliferation are implicated in the therapeutic action of ZHAST in treating OSAHS. Research indicates that hypoxic responses are a prominent feature of OSAHS, contributing significantly to its pathophysiology. Within the context of OSAHS, protein phosphorylation plays a critical role in mediating cellular responses to intermittent hypoxia, which is a hallmark of this condition. Intermittent hypoxia can lead to oxidative stress and inflammatory responses involving the activation of signaling pathways dependent on the phosphorylation of key proteins (Yi et al., 2022). For instance, Zhao (2021) discusses how TNF-α promotes insulin resistance via the TNF-α/IKKβ/IKβ/NF-κB signaling pathway—a process heavily reliant on the phosphorylation state of these proteins (Zhao et al., 2021). This pathway example illustrates how phosphorylation influences inflammatory pathways potentially exacerbating metabolic disturbances in OSAHS. KEGG enrichment analysis suggests that the pharmacological mechanisms by which ZHAST treats OSAHS may primarily involve pathways such as cancer, hepatitis B, MAPK signaling, AGE-RAGE signaling in diabetic complications calcium signaling lipid and atherosclerosis neurotrophic signaling. The MAPK (Mitogen-Activated Protein Kinase) signaling pathway is a crucial mechanism in cellular physiology responsible for transmitting extracellular signals to mediate cellular responses to stress inflammation and developmental cues. This pathway involves activating a series of protein kinases that through phosphorylation activate transcription factors regulating gene expression thereby influencing cell growth differentiation and survival (Xu et al., 2021). Within the context of OSAHS the MAPK pathway plays a crucial role in responding to intermittent hypoxia—a hall.

## 5 Conclusion

In this study, we explored the therapeutic mechanisms of the traditional Chinese medicine formula ZHAST for OSAHS using network pharmacology and molecular docking techniques.Our findings demonstrate that the active components in ZHAST interact with multiple key targets, influencing the pathophysiological processes of OSAHS. Particularly, by modulating signaling pathways related to hypoxia response and protein phosphorylation, it exhibits potential therapeutic effects. These discoveries provide a scientific basis for the use of ZHAST in treating OSAHS and unveil its potential therapeutic mechanisms. In summary, this research highlights the pharmacological substances and mechanisms of action of the traditional Chinese medicine formula ZHAST in treating OSAHS. Future studies may further explore additional active components and their mechanisms of action, providing a more robust scientific foundation for subsequent research. Although some important preliminary findings were obtained, this study still has some limitations. To investigate the involvement of ZHAST in the treatment of OSA, we used only advanced bioinformatics and computational techniques. Therefore, the reliability and accuracy of the predictions need to be further *in vivo* animal experiments verified.

## Data Availability

The raw data supporting the conclusions of this article will be made available by the authors, without undue reservation.
